# Through the Mouth or Nostrils: The Methane Excretion Route in Belching Dairy Cows

**DOI:** 10.3390/ani15162350

**Published:** 2025-08-11

**Authors:** Ligia Johana Jaimes, Sebastián Castrillón, Brandon Stiven Bustamante, Héctor Jairo Correa

**Affiliations:** 1 Medicine-Veterinary Facultity, Institución Universitaria Visión de las Américas, Medellín 050031, Colombia; 2 Agrarian Sciences Facultity, Universidad Nacional de Colombia, Medellín 050034, Colombia; secastrillonmo@unal.edu.co (S.C.); bsbustamantec@unal.edu.co (B.S.B.); 3 Animal Production Department, Agrarian Sciences Facultity, Universidad Nacional de Colombia, Medellín 050034, Colombia; hjcorreac@unal.edu.co

**Keywords:** data logger, exhalations, grazing, normal respiration

## Abstract

Methane is a potent greenhouse gas (GHG) emitted from several anthropogenic sources, most notably enteric fermentation in domestic ruminants, primarily during exhalation. To date, however, it is unclear whether the excretion route of methane exhaled by ruminants occurs through the mouth or nostrils and what the pattern of excretion is; this is important in designing equipment and methods to measure the methane emitted by ruminants. Thus, the objective of this experiment was to quantify the exhaled methane excreted by dairy cows via the nostrils and mouth while resting, grazing, and ruminating, as well as the pattern of concentration during and between belching. Four tests were conducted in this study with four dairy cows each. In the first test, resting cows, carrying an electronic spirometry mask (ESM) assembled with a methane sensor in its outlet, were used to measure the concentration of methane in the air expelled through the nostrils and to model the methane concentration between belches; in the second test, a mouth mask was used to measure methane excreted through the mouth; in the third test, an ESM with a methane sensor assembled over the mouth was used to measure methane excreted through the mouth; finally, in the fourth test, a methane sensor was manually placed at 2.0–3.0 cm in front of the mouth of ruminating cows to measure the methane concentration. In the first test, a pattern of methane concentration between belches was identified, as well as a pattern of the methane concentration and volume of air exhaled during belching. Methane excretion through the mouth was not detected in any of the tests; hence, it is concluded that dairy cows, while resting, ruminating, and grazing, emit enteric methane only through the nostrils under normal respiration conditions. This is important in the respiratory physiology of ruminants and in designing equipment and methodology to measure cranial methane excretion.

## Simple Summary

The objective of this study was to investigate the cranial routes of methane evacuation during eructation in dairy cows while resting, ruminating, and grazing and to develop a mathematical model of the pattern of methane concentration between belches. It was determined that methane exits only via the nostrils under normal respiration conditions.

## 1. Introduction

Methane is a potent greenhouse gas (GHG) emitted by natural and anthropogenic sources, with enteric fermentation in domestic ruminants being one of the most significant anthropogenic sources globally [1]. In Colombia, based on Tier 2 of the IPCC, it is estimated that 24.3% of the total GHG emissions consist of methane, with 52.02% of this gas produced from enteric fermentation in cattle [2]. This estimation has, however, an uncertainty of 11.6%, and to reduce this uncertainty and improve the precision of GHG emissions, research is needed to generate useful information for Tier 3 of the IPCC. For this purpose, it is necessary to measure the methane emission from the most direct source, which in the Colombian cattle case is the grazing condition, as this is the most important production system [3]. Several techniques used to measure cranial methane emissions by grazing animals have recently been reviewed [4]. However, there is confusion about the mechanisms and routes of methane excretion in ruminants, complicating the design and utilization of equipment, as well as the development of methodologies for appropriate methane emission measurement.

Exhalation is the most important mechanism of excretion of methane in ruminants. Although Reiset [5] noted that methane production during exhalation in ruminants was associated with the ruminal fermentation of plant foods, the author did not describe the mechanism linking these two phenomena. Currently, two mechanisms that explain this relationship are known: first, the absorption of enteric gases through gastrointestinal epithelium, initially described by Tappeiner [6] and then quantified by Murray et al. [7] and Reynolds et al. [8]; and, second, the evacuation of ruminal gases through belching, which are inhaled into the lungs when the nasopharyngeal sphincter closes in the final stage of belching. This phenomenon was accidentally discovered by Colvin et al. [9] when observing a steer with a tracheal fistula, in which rumen gas escaped from around the cannula at the time of eructation. Hoernicke et al. [10] confirmed this finding and improved the data on the quantity of gases eructed and the methane concentration; however, it was Dougherty et al. [11] who established the importance of the nasopharyngeal sphincter in preventing the expulsion of eructed gases through the nose. Thus, the gases eructed are mixed in the lungs with atmospheric ones after each inhalation and with the carbon dioxide produced from cellular metabolism. Therefore, since exhalation is the mechanism of evacuation for gases mixed in the lungs, it is also the most important mechanism of excretion for enteric gases in ruminants, including methane. However, it is not clear whether these gases are exhaled through the nostrils, through the mouth, or through both routes; ruminants are capable of each of these, depending on the physiological state of the animal during respiratory activity [12]. Thus, while Sorg [13] declared that the gas eructed from the rumen is first inhaled into the lungs and then excreted through exhalation via nostrils, Murray et al. [7], Palangi et al. [14], and Glasson et al. [15] claimed that most of the methane produced in rumen is excreted by belching and then evacuated through the mouth. Others have also suggested that belching gases travel through both the mouth and nostrils [16,17], but without any evidence. This implies that there is still confusion about the physiology of belching; therefore, this uncertainty affects the design and utilization of equipment, as well as the development of methodologies to appropriately measure cranial methane emission in ruminants. This has prevented the generation of sufficiently detailed information to understand, among other aspects, the pattern of cranial methane emission in these animals. Studies have been published that reveal changes in the methane concentration during and between belches. Ricci et al. [18] and Sorg et al. [19], using a laser methane detector, described a rapid increase in methane concentration during eructation followed by mini-peak and mini-valley values, while Koch et al. [20] showed a graph of the variation in methane concentration between belches, also indicating a rapid increase in this concentration after eructation and a variation during the decrease in the methane curve as a product of pulmonary exhalation and inspiration. However, these variations do not show a consistent pattern and, therefore, have not been mathematically modeled. Thus, the aim of this study was to quantify the relative excretion of methane through the nostrils and the mouth in dairy cows resting, grazing, and ruminating under normal respiratory conditions and the pattern of concentration during and between belching, on the hypothesis, that under these conditions, cows exhale only through the nostrils with a distinctive pattern.

## 2. Materials and Methods

The study was divided into four tests and was conducted in January 2024 at the Universidad Nacional de Colombia Agrarian Station Paysandú, in Santa Elena (Medellín), located at 2500 m of altitude, considered a highly humid tropical climate. There, animals graze on Kikuyu grass (*Cenchrus clandestinus*) and are supplemented with concentrated food during milking. The Ethics Committee in Animal Research of Universidad Nacional de Colombia at Medellín (Antioquia) approved this study as part of the research projects HERMES 57859, “Evaluation of an equipment to measure the air exhaled by Holstein cows” and HERMES 57438, “Interfaculty alliance for the reduction of greenhouse gases in cattle farming”.

**Figure 1:**
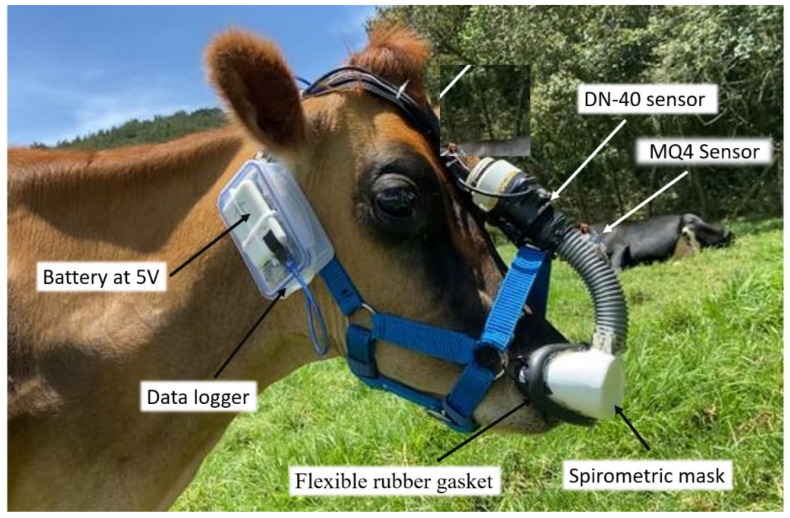
An experimental cow with an electronic face mask with an MQ4 sensor, used to measure the methane excreted through the nostrils in Test 1. Source: authors.

This was an exploratory study, due to insufficient prior empirical information on the exhaled air pathway in ruminants, and was based on a limited number of experimental articles on their eructation and respiration physiology in which a small number of experimental animals were used [7,8,9]. In the first test, four dairy cows, resting and carrying an electronic spirometry mask (ESM), covering only the nostrils and assembled with a high sensitivity to methane semiconductor sensor (MQ4) (Hanwei Electronics, Zhengzhou, China), were used to measure the concentration of methane in the air expelled through the nostrils (Figure 1). The design of the ESM was based on the spirometry mask described by Correa and Jaimes [16] with some modifications (Appendix A) that included an electronic circuit (Appendix B). Programming code was written in ARDUINO IDE 2.2.1 to record data each 200 ms in a micro-SD module (Appendix C).

The information gathered from this test was used to analyze the belching pattern and to model the concentration of methane exhaled when belching. To accomplish this, several mathematical models were revised, and a sinusoidal function (with two coefficients) superimposed on the Wood model [21] and modified (with four coefficients) was chosen to estimate this variable, minimizing the square error of the area under curve of the methane concentration. The model used was

CH4, ppm = (SINE(a*t)*c/√t) + (d + e*t^f*n(−g*t)),

where a = coefficient that establishes the number of exhalations; t = time (s); c = amplitude of the methane concentration variation; d = initial methane concentration; e = increased methane concentration; f controls the increase in methane concentration; n = neper number (2.71828); g controls the decrease in methane concentration. The model parameters were estimated by nonlinear least squares using the PROC NLIN procedure in SAS version 9.4 (Appendix D).

**Figure 2:**
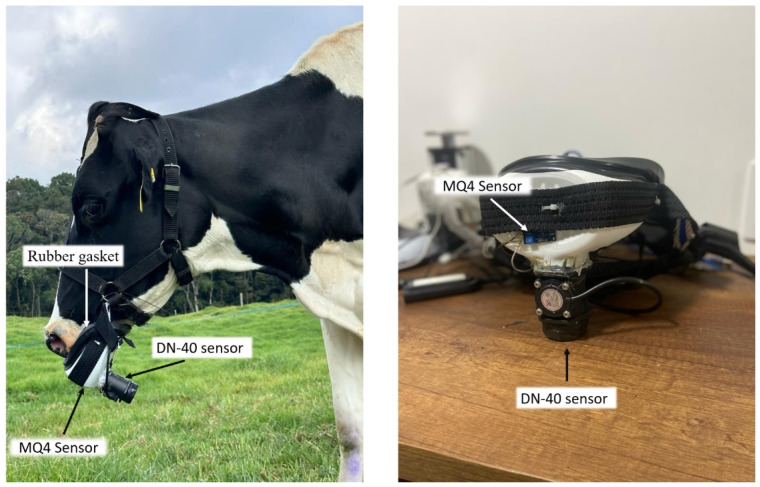
An experimental cow with a mouth mask in test 2. As can be seen in the image on the right, the mask only covers the animal’s mouth from the upper jaw down, leaving the nostrils free. The airflow sensor (DN-40) can also be seen, as well as the location of the methane sensor (MQ-4), mounted inside the mask. The image on the left shows the location of the methane sensor more clearly. Source: authors.

In the second test, a mouth mask was used to quantify the total gases and methane concentration belched and excreted through the mouth by another four dairy cows resting and ruminating. A methane sensor MQ4 was assembled inside the mouth mask, and at the outlet, a flowmeter sensor DN-40 was connected to a data logger (Figure 2). Additionally, visual observations of the cow’s mouth while resting without the mouth mask were recorded to confirm or discard the opening of the mouth and evacuation of gases during belching.

**Figure 3:**
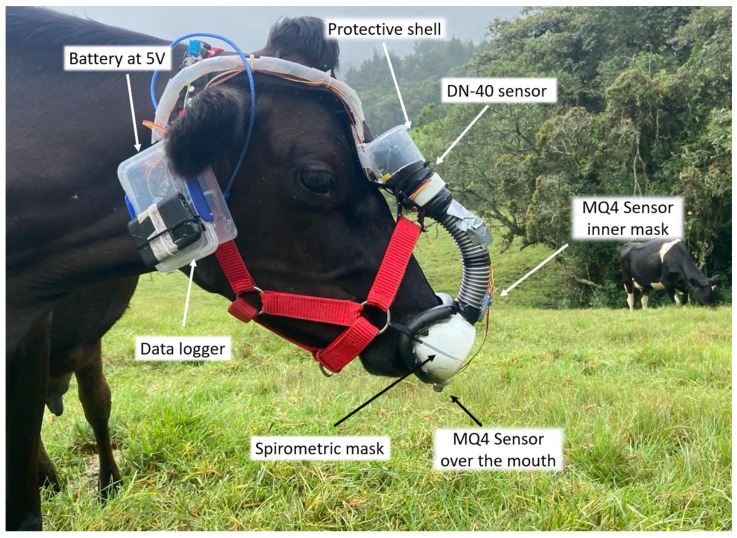
An experimental cow wearing the electronic face mask with an MQ4 sensor to measure the methane excreted through the nostrils and another to measure the methane excreted through the mouth in test 3. Source: authors.

**Figure 4:**
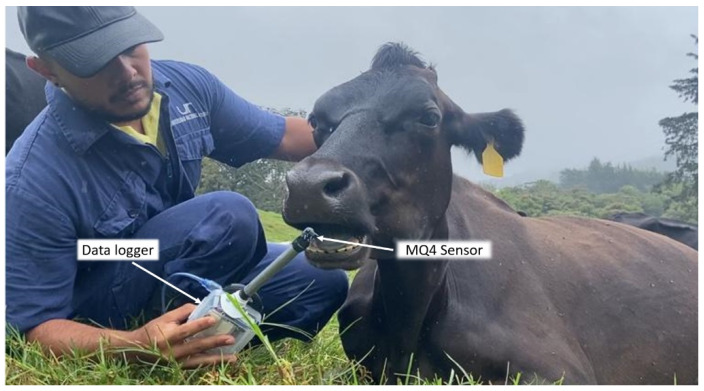
Sensing methane concentration from the mouth of an experimental cow ruminating using the MQ4 sensor in test 4. Source: authors.

In the third test, an ESM was used with a second methane sensor assembled over the mouth to confirm or deny the evacuation of methane through the mouth in four cows resting, ruminating, and grazing (Figure 3). Finally, in the fourth test, a methane sensor connected to a data logger was manually placed at 2.0–3.0 cm in front of the mouth of four ruminating cows to measure the methane concentration during and between belching (Figure 4). All tests were conducted for 5 to 15 min per cow, registering between 1300 and 4500 data points of methane concentration and air flow/cow. This time is used in spot sampling techniques to measure methane emissions [4], ensuring that at least three belches are recorded [22], if one takes into account that the mean time for the interval between belches is between 54 [23] and 64 s [22].

The methane concentration was expressed in ppm, and the volume of evacuated air was expressed in L/min. In the first, second, and fourth tests, data were graphed, and the identification of peaks associated with eructs was performed using the FINDPEAKS function of the MATLAB software (version R2023b, The MathWorks, Inc., Natick, MA, USA). When two or more peaks were localized at less than 5 s, they were considered as part of the same eruct. In the third test, the proportion of methane exhaled via the nostrils and mouth was quantified and analyzed using the McNemar test for paired samples with the statistical software SAS version 9.4.

## 3. Results

**Figure 5:**
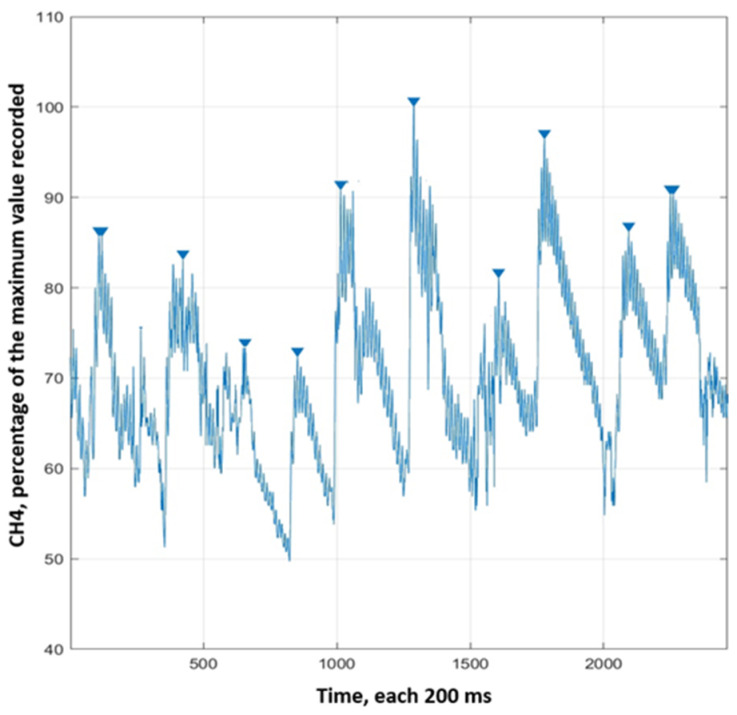
An example of methane data analysis to identify belching in test 1, using MATLAB. The methane data are expressed as a percentage of the maximum value recorded. The abscissa axis shows the data registered each 200 ms. Source: authors.

Test 1 clearly identified belching in all the experimental cows. Figure 5 shows an example of the methane data analysis, using the FINDPEAKS function in MATLAB to identify belching. These results indicate that methane is evacuated through the nostrils during exhalations from the lungs.

**Figure 6:**
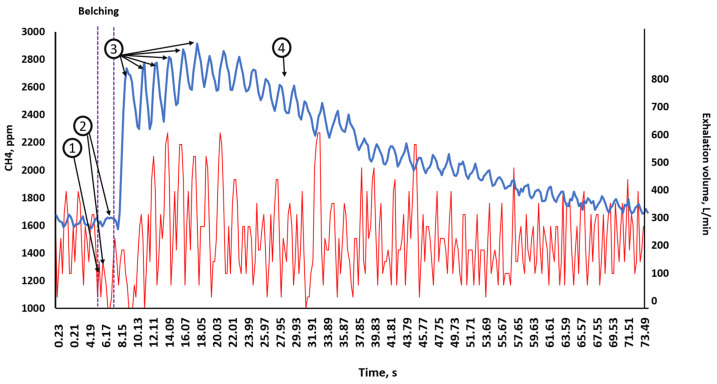
Methane concentration pattern (blue line) and exhaled air volume (red line) between belches. Detailed recorded phenomena are highlighted: 1. inhalation stops, and a belch is heard; 2. a brief exhalation is observed with a slight increase in methane concentration; 3. then, the methane concentration increases over the next four to six exhalations; 4. finally, the methane concentration gradually decreases with each exhalation until the next belch occurs. Source: authors.

**Figure 7:**
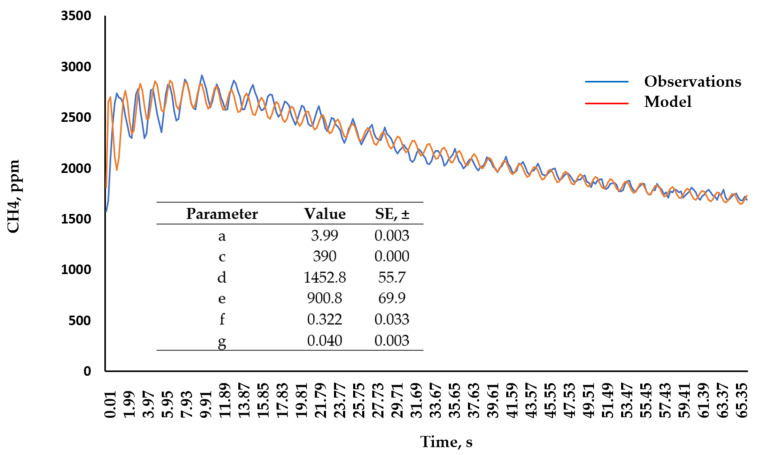
Parameters of the model to estimate the methane concentration during exhalations between two belches (CH4, ppm = (SINE(a*t*c)/√t) + (d + e*t^f*e(−g*t)), *p* < 0.001; r2 = 0.873; RMS = 94.3 ppm). Source: authors.

Figure 6 shows an example of the methane concentration pattern between the belches and the exhaled air volume, highlighting the ESM-recorded phenomena that occur during a belch. The inter-belch methane concentration shown in Figure 6 has a distinctive pattern that has not been previously described, which was modeled using a sinusoidal function superimposed on the modified Wood model, as shown in Figure 7. This mathematical model minimized the square error of prediction and the square error of the area under the curve of the methane concentration. The model shows an initial increase (from 1572 ppm) to a maximum value at the sixth exhalation (2916 ppm) that was 0.85 times higher; after this, a decrease in concentration was observed during the following exhalations until reaching a minimum before the next burp (1719 ppm). Now, by integrating the CH4 concentration data (mean: 2199 ppm) with the volume of air excreted in exhalations (tidal volume) (mean: 3.72 ± 1.89 L/exhalation) (n = 44) between the two burps presented in Figure 6, we calculated that the amount of methane emitted between the two burps was 0.363 L, with an interval between belching of 66 s and a rate of emission of 0.328 L/min (equivalent to 0.311 kg/min based on a density of 0.217 kg/m^3^, 1 psi of pressure, and 18 °C).

**Figure 8:**
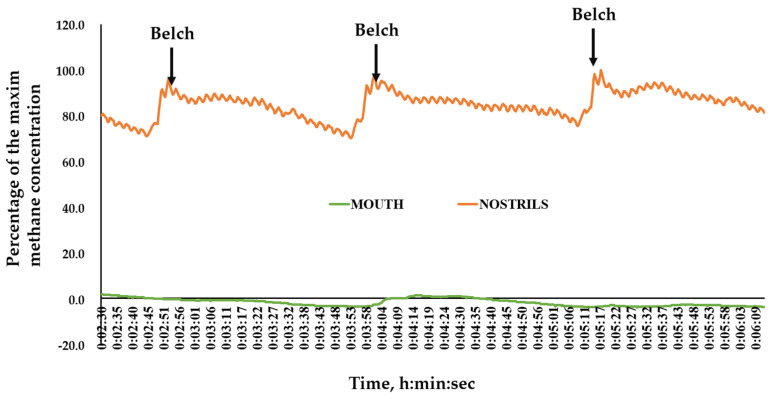
An example of the data obtained in test 3 using a spirometry mask with an MQ4 sensor assembled above the mouth. Data are expressed as percentage of the maximum value registered. Source: authors.

**Scheme 1:**
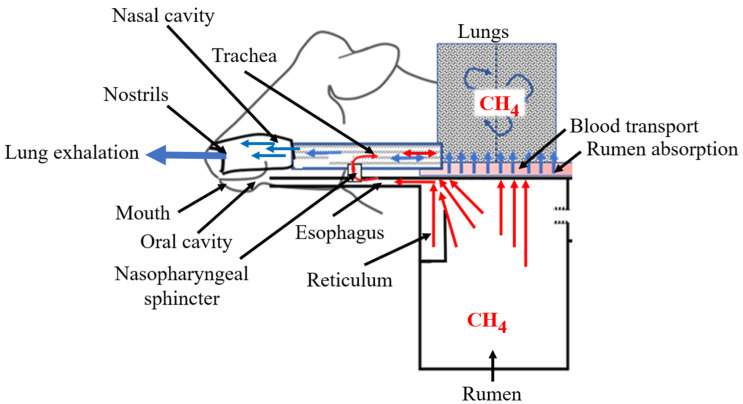
Schematic routes of the cranial evacuation of ruminal methane in animals under normal respiratory conditions. According to the literature reviewed and the data analyzed in this experiment, rumen methane is partially absorbed and transported by blood to the lungs [8]. Unabsorbed methane is belched and then deviated to the lungs through the trachea when the nasopharyngeal sphincter closes, the glottis is opened, and respiration stops [24]. Gases in the lungs are evacuated by the nostrils during each exhalation; thus, all the rumen methane exits through the nostrils during exhalations and belching. Source: authors.

The results of test 2 were negative for methane, indicating that this gas is not evacuated through the mouth in cows resting and ruminating. A modified spirometry mask was used in the third test: a second MQ4 sensor was assembled to measure the methane evacuation through both the nostrils and mouth simultaneously. However, methane was not found to be exhaled through the mouth (Figure 8). The slope of the regression equation using data registered by the methane sensor on mouth in this test (Y = −0.0041x + 3 × 10^−5^, r2 = 0.375) suggests that the concentration of methane detected by this sensor does not change with belching. Although a test was not designed to determine whether air leaks occurred with the mask, the results of this third test suggest an adequate function of the mask, with no evidence of methane concentrations found either at the mouth or through the packaging with which the methane sensor is mounted. Finally, test 4 also showed there was no methane evacuation through the mouth in ruminating cows. Based on these results, a scheme representing the route of cranial methane evacuation in ruminants resting, grazing, and ruminating is proposed (Scheme 1).

## 4. Discussion

The hypothesis of this study was that under normal respiratory conditions dairy cows grazing, resting, and ruminating exhale enteric methane only via the nostrils. The results suggest that this is correct.

With ESM, the animals are able to consume meadow grasses, drink water, and breathe without difficulty, as reported by Correa and Jaimes [16] suggesting that the animals were not under stress during the experiments. Moreover, this equipment has the additional advantage of being cheap and easy to assemble, it measures the volume of air exhaled and the frequency and duration of exhalations–inhalations, and the animals adapt quickly to it [16]. However, a critical point in the operation of the ESM is guaranteeing the airtightness of the packaging with the flexible rubber gasket. This allowed us to obtain the results presented here.

The cessation of inhalation, observed in Figure 6, when belching occurs is due to the closure of the nasopharyngeal sphincter at the moment when the gas bag from the rumen moves through the esophagus and reaches the posterior part of the pharynx. At that moment, the glottis opens and, consequently, breathing stops to allow the gas bag to enter the trachea and penetrate deeply into the lungs as described by Dougherty [24]. However, there is no consensus about the percentage of belching gases that enters the lungs and the percentage that is exhaled directly from belching. Thus, while Hoernicke et al. [10]—using two tracheostomized Jersey cows and an isotope tracer method—reported that between 71 and 99% of belched gas were exhaled through the trachea, Murray et al. [7], on the other hand—using two rumen cannulated sheep with face mask and an isotope tracer method—calculated that only 5% of rumen methane was excreted through the lungs. However, these data may be due to an error in the methodology used to measure the methane absorbed by the ruminal epithelium, which affects the concentration of methane in the rumen gas and, subsequently, the methane evacuated by belching. To avoid belching, the authors allowed rumen gas to escape through an open cannula; however, this method reduced the concentration gradient between the rumen gas and blood and resulted in low methane absorption, as previously established by Hoernicke et al. [10]. Similarly, Murray et al. [7] reported considerable methane loss through the rumen cannula, even though precautions were taken to minimize it, which also affected the methane absorption. Recently, Reynolds et al. [8] measured the methane concentration in the portal veins of two Holstein lactating cows and estimated that rumen methane absorption could represent 12% of total rumen methane production. The data shown in Figure 6 indicate that methane excretion during belching is lower than the excretion during the seconds before and after belching. Thus, as an example, methane excretion (the concentration of methane multiplied by the volume of exhaled air divided by a million) during the first belch, shown in Figure 6, was 37.2% lower than the mean excretion six seconds before and 66.7% lower than methane excretion six seconds after belching. In addition, the mean methane concentration in the air exhaled by the four cows (1955 ± 155 ppm) was nearly 160 times lower than the published methane concentration in rumen (306,944 ± 54,865 ppm) reported by Moate et al. [25] and Washburn and Brody et al. [26] (317,264 ± 61,092 ppm), indicating the dilution of this gas in the lungs before its evacuation through the nostrils and no effect of belching on the methane concentration in the exhaled air.

The methane concentration values in the exhalations found in this work, shown in Figure 6 and Figure 7, are similar to the values reported by Washburn and Brody et al. [26], who used face masks that completely covered the snout of lactating cows. However, these values are higher than those reported by the same authors using a non-lactating jersey cow, as well as the concentrations measured by Koch et al. [20] in air exhaled by four non-lactating Jersey cows using an electro-chemical sensor placed underneath a nose-screen above the nostrils. These differences may be due to the fact that the experimental cows in this research were lactating, and there is a known positive relationship between the milk production level and the methane concentration in exhaled air [27]. These concentrations are also higher than the values published by other researchers using different methods. Thus, Antanaitis et al. [28] found a CH4 concentration in the exhaled air of Holstein dairy cows between 187.6 and 348.6 ppm using a laser methane detector, while Sypniewski et al. [29] reported a maximum CH4 concentration of 1000 ppm using the sniffer method, and Huhtanen et al. [30] reported values between 330 and 1750 ppm using Greenfeed equipment.

The mean tidal volume of the data shown in Figure 6 (3.72 ± 1.89 L) is within the minimum and maximum values expected for dairy cows [31] and is similar to that calculated from the tidal volume equivalent to 8.0 ± 1.4 mL/kg of body weight [32], suggesting that the flow sensor assembled in the electronic mask used in this experiment and its calibration satisfactorily measured the tidal volume of dairy cows.

Experiments on the physiology of belching in ruminants are scarce. Although recent authors have claimed that methane can exit through the mouth during belching [14,15], this affirmation is not based on experimental evidence. In turn, others claim that methane can exit through the nostrils and mouth during belching [33,34], also without evidence. Although Murray et al. [7] used an isotope tracer method to estimate methane production in sheep, their methodology did not distinguish between the evacuation of this gas through the mouth and through the nostrils during belching, since they used a fiberglass mask placed over the muzzle of the sheep. Previously, Colvin et al. [9], when studying belched gases in ruminants, used a face mask over the muzzle of animals to prevent the discrimination between belched gases’ evacuation through the nostrils and through the mouth. Thus, no experimental publication was found that measured the evacuation of enteric methane in ruminants distinguishing between the mouth and nostrils. This experiment demonstrates for the first time that, under normal conditions, enteric methane in ruminants is evacuated only through the nostrils and not through the mouth. This may be because, as in other mammals, ruminants can exhale air through the mouth only during episodes of tachypnea (heat stress, respiratory disease, strenuous exercise, etc.) [12]. Therefore, the nostrils can be expected to be the main route of exhaled air [35,36] and, thus, enteric methane during normal respiration—as was the case in this experiment. These findings are important for designing equipment and methods to appropriately measure methane emissions in ruminants.

## 5. Conclusions

With the methodology and equipment used in each test of this study, we did not find evidence for the evacuation of gases and methane via the mouth during eructation in dairy cows while resting, grazing, and ruminating, suggesting that all rumen methane is exhaled via the nostrils during the normal respiration of the animals. Likewise, for the first time, the pattern of methane concentration between belches was modeled. Thus, the electronic spirometry mask used in the first test is an appropriate piece of equipment for measuring the emissions of enteric methane in grazing cattle.

## Data Availability

Data are available upon request to the corresponding author.

## Appendix A. Description of Spirometry Mask Modification of the Original Mask Described by Correa and Jaimes [16]

The components used to collect and store exhaled air samples in the study of Correa and Jaimes [16] were replaced by a methane sensor assembled at the outlet of the mask. Additionally, a data logger based on an Arduino (model UNO R3 MEGA328P, Si Tai&SH, Shenzhen, China) was incorporated to record the data of the MQ4 and flowmeter sensors DN-40 (Louchen ZM, Changsha, China). The flowmeter sensor DN-40 was calibrated as described by Correa and Jaimes [16], and the MQ4 sensor was calibrated in two phases: first, according to the manufacturer’s instructions after a minimum 24 h of preheating time and, second, using a methane analyzer with an N55A 22YS catalytic combustion sensor (Nemoto Special Chemicals Co., Ltd., Tokyo, Japan) calibrated in a certified laboratory (Laboratorio MLS, Medellín, Colombia). The exhaled air, stored in bags during the calibration of the flowmeter, was used to calculate a regression curve between the data of the MQ4 sensor and methane analyzer.

## Appendix B. Circuit Diagram of Sensors’ Connection to the Arduino Data Logger Shield

The circuit diagram below shows the connection of parts:

## Appendix C. Arduino Code for the ESM

#include <SPI.h>
#include <SD.h>
#include <TimeLib.h>
#include <Wire.h>
#include "RTClib.h"

RTC_DS1307 rtc;
DateTime dateTime;

File file;
volatile int pulses = 0;
const int flowSensor = 2;
unsigned long totalPulses = 0;
unsigned int flowRate;
float totalLiters;
float methane;
void countPulses() {
pulses++;
}

void setup()
{
pinMode(flowSensor, INPUT);
Serial.begin(115200);
rtc.begin();
setTime(0, 0, 1, 29, 7, 2023);
attachInterrupt(digitalPinToInterrupt(flowSensor), countPulses, RISING);
interrupts();
Serial.print("Initializing SD card…");
if (!SD.begin(10)) {
Serial.println("Initialization failed!");
return;
}
Serial.println("Initialization successful.");
}

void loop() {
time_t t = now();
unsigned long myTime = millis();
file = SD.open("UNICORN.CSV", FILE_WRITE);
if (file) {
dateTime = rtc.now();
int h = dateTime.hour();
int m = dateTime.minute();
int s = dateTime.second();
int d = dateTime.day();
int mo = dateTime.month();
int y = dateTime.year();

Serial.print("hour = ");
Serial.print(h);
Serial.print(":");
Serial.print(m);
Serial.print(":");
Serial.print(s);
Serial.print(":");
Serial.print(d);
Serial.print("/");
Serial.print(mo);
Serial.print("/");
Serial.print(y);
Serial.println("/");

file.print("hour = ");
file.print(h);
file.print(":");
file.print(m);
file.print(":");
file.print(s);
file.print(":");
file.print(d);
file.print("/");
file.print(mo);
file.print("/");
file.print(y);
file.println("/");

int sensorValue = analogRead(A1);
// print out the value you read:
Serial.print("CH4: ");
Serial.println((sensorValue-454)*3.3);// The values are an example after calibration
file.print("CH4: ");
file.println((sensorValue-454)*3.3);

totalPulses += pulses;
flowRate = (pulses * 2.7 / 0.085);// The values are an example after calibration

pulses = 0;

totalLiters = totalPulses * 2.7 / 27;

Serial.print(flowRate);
Serial.print(" L/hour");
file.print(flowRate);
file.print(" L/hour");

Serial.println(millis());
file.println(millis());

while(millis() < myTime+10);
file.close();
} else {
Serial.println("Error opening file.");
}

}

## Appendix D. NLIN Procedure in SAS/STAT for Methane Concentration

data SINE;
options pagesize=500;
options linesize=120 nocenter;
title 'NONLINEAR REGRESSION';
input t ppm;
cards;

PROC NLIN DATA=SINE;
PARMS A=4.0 C=390 d=1400 e=870 f=0.3 g=0.01;
MODEL ppm = SIN(a*t*c)/SQRT(t)+(d+e*t**f*2.71828**(-g*t));
RUN;
